# Fatty acid binding protein 4 induces osteogenesis and angiogenesis as pathogenesis of metabolic osteoarthritis

**DOI:** 10.1186/s10020-025-01330-2

**Published:** 2025-12-19

**Authors:** Chaofan Zhang, Yinjun Mao, Yishan Xin, Hongyan Li, Maocan Cai, Yiming Lin, Xuehui Zhang, Ying Huang, Yang Chen, Zida Huang, Xinyu Fang, Wenming Zhang, Yunzhi Lin

**Affiliations:** 1 Department of Orthopaedic Surgery, The First Affiliated Hospital, Fujian Medical University, 20 Chazhong Rd, Fuzhou, 350005 China; 2 Department of Orthopaedic Surgery, National Regional Medical Center, Binhai Campus of the First Affiliated Hospital, Fujian Medical University, Fuzhou, 350212 China; 3 Fujian Orthopaedics Research Institute, the First Affiliated Hospital, Fujian Medical University, Fuzhou, 350005 China; 4 Fujian Orthopedic Bone and Joint Disease and Sports Rehabilitation Clinical Medical Research Center, the First Affiliated Hospital, Fujian Medical University, Fuzhou, China; 5 Department of Pharmacy, The First Affiliated Hospital of Fujian Medical University, Fuzhou, 350005 China; 6 Department of Pharmacy, National Regional Medical Center, Binhai Campus of the First Affiliated Hospital, Fujian Medical University, Fuzhou, 350212 China; 7 School of Health Management, Fujian Medical University, Fuzhou, 350005 China; 8 Department of Stomatology, The First Affiliated Hospital, Fujian Medical University, 20 Chazhong Rd, Fuzhou, 350005 China; 9 Department of Stomatology, National Regional Medical Center, Binhai Campus of the First Affiliated Hospital, Fujian Medical University, Fuzhou, 350212 China

**Keywords:** Osteoarthritis, Fatty acid binding protein 4 (FABP4), Subchondral bone, Bone remodeling, Angiogenesis

## Abstract

**Background:**

The pathogenesis of osteoarthritis (OA) is not yet fully elucidated. FABP4 plays a role in the occurrence of metabolic OA, however, the mechanism remains unclear. The purpose of this study was to further explore the mechanism by which FABP4 mediates the occurrence of metabolic OA.

**Methods:**

In vivo, FABP4 knockout mice (KO) and wild-type littermates (WT) were fed with high-fat diet (HFD) for 3 and 6 months. WT mice were fed with HFD and treated with FABP4 inhibitor BMS309403 (30 mg/kg/d) or vehicle for 6 months. Knee cartilage degenerative changes and subchondral bone changes were assessed. In vitro, FABP4 was used to stimulate mouse bone marrow mesenchymal stem cells (mMSCs) and endothelial progenitor cells (EPCs). Osteogenesis and angiogenesis were assessed.

**Results:**

In vivo, knocking out of FABP4 and pharmaceutical inhibition of FABP4 significantly alleviated subchondral bone sclerosis and type H vessel formation in mice fed with HFD, and was significantly associated with osteogenesis and angiogenesis. In vitro, FABP4 promotes the differentiation of MSCs into osteoblasts through activation of the PI3K/Akt signaling pathway, and promotes the expression of osteogenesis-related proteins. FABP4 also promotes endothelial cell migration, tube formation, and wound healing through activating the PI3K/Akt pathway.

**Conclusions:**

This study suggests that FABP4 induced subchondral bone osteogenesis and angiogenesis. The PI3K-Akt signaling pathway plays a critical role in both processes. Inhibition of FABP4 may serve as a potential therapeutic approach for metabolic OA.

**Supplementary Information:**

The online version contains supplementary material available at 10.1186/s10020-025-01330-2.

## Introduction

Osteoarthritis (OA) is a worldwide highly prevalent chronic joint disease that causes pain, disability, and loss of function (Long et al. 2022). OA is a disease of the whole joint, involving cartilage degradation, bone remodeling, osteophyte formation, joint inflammation, and loss of normal joint function (Kraus et al. 2015). It was estimated that 242 million people were living with symptomatic and activity-limiting OA of the hip and/or knee (Syx et al. 2018). However, no current drug is able to modify the progression of OA and prevent long-term disability (Latourte et al. 2020). A better understanding of the causes of the disease is essential for formulating prevention and treatment strategies.

OA is a multifactorial disease. Intrinsic and extrinsic risk factors are attributable to the pathogenesis of OA, including congenital defects, aging, gender, trauma, and obesity (Tu et al. 2019). Obesity is a common metabolic syndrome caused by excess fat accumulation in the body due to disorders of fat metabolism (Natesan and Kim 2021). Previous research has demonstrated a strong association between knee OA and obesity (Xie and Chen 2019). Previously, it is well-recognized that obese individuals are more vulnerable to suffer from OA due to excessive mechanical stress on weight-bearing joints, such as hip and knee. However, recent studies have shown that obesity also contributes to joint degeneration by producing and releasing a plethora of factors termed adipokines (Gómez et al. 2011). Studies in the past two decades have shown that adipokines, including leptin, adiponectin (Gómez et al. 2011), resistin (Gómez et al. 2011), and visfatin (Gosset et al. 2008), play key roles in obesity-mediated OA (Xie and Chen 2019). However, the effect of adipokines on subchondral bone remodeling is still controversial. Some studies have shown that leptin can promote osteoblast proliferation and differentiation (Scotece et al. 2014). While both positive and negative effects of leptin on bone mass have been observed in another in vivo study (Neumann et al. 2016). For adiponectin, its role in subchondral bone is also unclear. Some studies have shown that adiponectin can promote osteogenesis and inhibit osteoclasts (Neumann et al. 2016).

Fatty acid binding protein 4 (FABP4) is a novel adipokine that can reversibly bind to hydrophobic ligands such as saturated and unsaturated long-chain fatty acids (FAs), eicosanoids, and other lipids, taking part in the regulation of lipid trafficking and responses at the cellular level (Rodríguez-Calvo et al. 2017). FABP4 expression is mainly expressed in adipocytes, but can also be detected in human endothelium of the placenta and placental trophoblast cells and is dispensable for maternal–fetal exchange and fetal growth. Emerging evidence suggests that FABP4 performs a vital role at the fetoplacental interface, involving maternal–fetal immune tolerance and lipid transportation (Shi et al. 2023). Coupling its impact on localized immunological aberrations, it plays a crucial role in pregnancy establishment and maintenance and further impacts fetal growth and neonatal size (Shi et al. 2023).

However, recent studies have shown that a high level of FABP4 in human blood was significantly positively associated with various metabolic diseases, including diabetes, insulin resistance, and atherosclerosis (Rodríguez-Calvo et al. 2017; Furuhashi 2019; Furuhashi et al. 2007). The latest research also demonstrates that FABP4, similar to other adipokines, plays an important role in the occurrence and development of OA (Zhang et al. 2018a, 2018b; Schadler et al. 2021). Our clinical data showed that the level of FABP4 in the plasma and joint fluid of patients with OA was significantly higher than that of patients without OA (Zhang et al. 2018b). FABP4 was also found to be negatively correlated with cartilage thickness (Schadler et al. 2021). In our previous studies, it was also demonstrated that knocking out or pharmacologically inhibiting FABP4 in mice can significantly alleviate cartilage degeneration (Zhang et al. 2018a). We further performed a mechanistic study and showed that FABP4 induces chondrocyte degeneration via activation of the NF-κb signaling pathway (Zhang et al. 2024a). Bioinformatics data revealed that FABP4 is observed in the brown module (a cluster of highly interconnected genes identified through weighted gene co-expression network analysis (WGCNA)) and protein–protein interaction (PPI) network associated with OA tissue and is involved in energy metabolism in subchondral bone cells (Gu et al. 2019). This data suggests that FABP4 induced OA, particularly metabolic OA.

However, the specific mechanism by which FABP4 mediates the occurrence of metabolic OA has not yet been elucidated. Our data also demonstrated that FABP4 has effects on chondrocytes in a dose-dependent manner (Zhang et al. 2018a). However, the role of FABP4 in subchondral bone is still not clear. We have also shown that FABP4 wild-type mice have a stronger degree of subchondral bone sclerosis than the FABP4 knock-out mice (Zhang et al. 2018a). Some studies have demonstrated that FABP4 can inhibit osteoclast activity: upregulation of FABP4 expression suppressed osteoclast activity, whereas inhibition of FABP4 expression promoted osteoclast activity (Song et al. 2018). However, other reports have demonstrated a negative correlation between FABP4 expression and the osteogenic marker ALP (Xu et al. 2021). There are also studies indicating that FABP4 may also promote vascular regeneration in rheumatoid arthritis (RA) (Guo et al. 2022). However, it is unclear whether FABP4 has a role in angiogenesis in metabolic OA and how FABP4 affects the process of bone remodeling.

Therefore, the purpose of this study is to explore the role of FABP4 in bone remodeling and angiogenesis. A high-fat diet (HFD)-induced obesity animal model was used in the study, as previous studies have proved that HFD in mice, which could significantly induce obesity, can induce OA (Griffin et al. 2010, 2012; Barboza et al. 2017; Donovan et al. 2018). In one study, a very high-fat diet (60% of kcals from fat) induces knee OA in association with increased adiposity, systemic pro-inflammatory mediators, and glucose intolerance (Griffin et al. 2012). It significantly increased OA in the medial femur and tibia compared to control animals. Thus, an HFD seems to induce or exacerbate the progression of OA in mice. The metabolic changes and systemic inflammation brought about by an HFD appear to be key players in the onset and progression of OA (Sansone et al. 2019). On the other hand, HFD has been widely proven to significantly elevate the circulating FABP4 level (Shu et al. 2017).

## Methods

### Ethics and animals

All experimental protocols were approved by the ethics committee of our institution (FJMU IACUC 2021–0384). FABP4 knockout (KO) mice in C57BL/6N background were generated as previously described (Zhang et al. 2018a). A total of 32 KO and their littermates (WT) mice (All males, ages 6 weeks) were used. Two parallel studies were performed. In the first study, weight-matched KO and WT mice (n = 32 each, around 18.0 g) were included. Animals were randomly allocated to 2 diet groups (n = 16 each): a high-fat diet group (HFD, 60% kcal from fat, 20% from carbohydrate, and 20% from protein. D12492, Research Diet, NJ, USA) and normal-diet group (ND, 11.6% kcal from fat, 52.9% from carbohydrate, and 20% from protein. 5053, LabDiet, Brentwood, MO, USA). As 60% HFD (Griffin et al. 2012) showed more consistent gains in body weight, body fat, and associated joint degeneration than the 45% HFD (Griffin et al. 2010), and thus was selected in the current study. In each group, the diet started from 6 weeks old (Zhang et al. 2018a; Barboza et al. 2017) and was sustained for 3 months and 6 months (n = 4 each). In the parallel study, 16 weight-matched WT mice were enrolled and were treated with daily oral gavage of BMS309403 (BMS) (FABP4 specific inhibitor, 30 mg/kg/d dissolved in PBS solution, Chemrenblock Technology, Jiangsu, China) or equivalent PBS solution (n = 4 each). In each group, treatment was sustained for 6 months. The dosage, preparation, and administration of BMS309403 were referred to in previous reports (Furuhashi et al. 2007; Zhang et al. 2018a; Hoo et al. 2013). Potential side effects of BMS309403 were strictly monitored during the whole study period.

### Histological analysis

Samples from mice were fixed in 4% paraformaldehyde, followed by decalcification in EDTA-buffered saline solution (pH 7.4, 0.25 M) for 21 days at 4 °C. Tissues were embedded in paraffin and then were cut longitudinally to obtain 5 μm sections. The evaluation of joint histopathological features was accomplished by HE staining, toluidine blue staining, safranin O-fast green staining, and Masson staining as per the manufacturer’s instructions. To evaluate the degree of cartilage deterioration, safranin O-fast green staining and toluidine blue staining images were applied for OARSI, cartilage degradation score, and characterizing relative cartilage thickness (Pritzker et al. 2006; Kwon et al. 2021). Imaging analyses were evaluated in a blinded manner. Sections were randomly assigned identification numbers, and two experienced investigators respectively assessed the slides.

### Immunohistochemistry staining

Immunohistochemistry was performed as previously described (Lin et al. 2022). Briefly, deparaffinized histological sections were incubated with rabbit monoclonal antibody to Osterix (ab209484; 1:100; Abcam, Cambridge, MA, USA) or VEGF (ab52917; 1:100; Abcam, Cambridge, MA, USA) overnight at 4 °C. The next day, slides were incubated with a second antibody (ab6721; 1:1000; produced by Abcam Ltd., Cambridge, UK) for 1 h, followed by a peroxidase-labeled streptavidin–biotin staining technique (DAB kit, Invitrogen, Paisley, UK).

### Immunofluorescence staining

For immunofluorescence staining, we used rabbit anti-osteocalcin antibody (ab93876, 1:100, Abcam, Cambridge, MA, USA) as the primary antibody for osteogenesis to incubate sections at 4 °C overnight. Rabbit anti-CD31 antibody (ab281583, 1:100, Abcam, Cambridge, MA, USA) and rat anti-endomucin (ab106100; 1:100; Abcam, Cambridge, MA, USA) were selected as the primary antibody to mark H-vessels (Zhao et al. 2024). Rabbit anti-FABP4 antibody (ab92501, Abcam, Cambridge, MA, USA) was used for FABP4 staining in the joint.

Subsequently, the specimens were stained with Alexa Fluor 488-labeled goat anti-rabbit IgG H&L (ab150077, 1:1000, Abcam, Cambridge, MA, USA) or Alexa Fluor 594-labeled goat anti-rat IgG H&L (A-11007, 1:1000, Invitrogen, Paisley, UK) at room temperature for 1 h in the dark. Differentiated mMSCs were washed in PBS and fixed with 3.7% formaldehyde in PBS for 10 min at room temperature. Rabbit anti-osterix (ab209484; 1:100; Abcam, Cambridge, MA, USA) and rabbit anti-osteocalcin antibody (ab93876, 1:100, Abcam, Cambridge, MA, USA) were used as the primary antibody, and Alexa Fluor 488-labelled goat anti-rabbit IgG H&L (ab150077, 1:1000, Abcam, Cambridge, MA, USA) was used as the secondary antibody. Cells were incubated with DAPI (Life Technologies) and observed using a confocal microscope (Nikon A1R MP + multiphoton; Nikon Instruments Inc., Melville, NY, USA).

### Microcomputerized tomography (CT) analysis

Immediately after animals were euthanized, the bilateral knee joints were harvested and scanned in a high-resolution micro-CT system (Venus001, PINGSENG Healthcare, Kunshan, China) with 37 mm slice thickness and 294 threshold values. Then, 3D histo-morphometric analysis was produced using the auxiliary histo-morphometric software (PINGSENG Healthcare Ctvox, Shanghai). The subchondral bone of the tibia plateau was manually selected for analysis. The region of interest (ROI) was achieved manually slice by slice. The bone parameters including bone mineral density (BMD, mgHA/cm^3), bone volume percentage (BV/TV, %), trabecular thickness (Tb. Th, mm), trabecular number (Tb. N, 1/mm), and trabecular separation (Tb. Sp, mm) were calculated.

### Cell culture

Mouse bone marrow mesenchymal stem cells (mBMSCs) from the bone marrow of C57BL/6 mice were purchased from Cyagen Biosciences Inc (China). mBMSCs were cultured and osteoblastic differentiation of MSC was carried out using osteoblastic induction medium (OIM) containing standard growth medium supplemented with 10^−8^ M Dex, 50 μg/ml ascorbic acid, and 10 mM glycerophosphate (Sigma-Aldrich, St Louis, USA). For each test, 3 Biological replicates were performed. Endothelial progenitor cells (EPCs) (Procell, CP-H162, China) were cultured in EPCs culture medium (EGM-2; Lonza) at 37 °C in a 5% CO_2_ incubator. To gain insights into the molecular mechanisms by which FABP4 promotes osteoblast differentiation, MSCs were starved for 5 h and stimulated with OIM or OIM + FABP4 (1000 ng/ml) for 15–60 min, before Western blotting (Binder et al. 2015; Lan et al. 2025). The cells were subjected to starvation treatment in order to synchronize them as much as possible within the same cell cycle. For each test, 3 Biological replicates were performed.

Primary bone marrow-derived macrophages (BMMs) were obtained from the long bones of 6-week-old C57BL/6 J mice. Specifically, cells were extracted from the femur and tibia bone marrow and cultured in a 100 mm dish containing complete α-MEM medium supplemented with 10 ng/mL M-CSF for 24 h. Non-adherent cells were then collected and cultured in fresh medium containing 50 ng/mL M-CSF. After 3 days, adherent cells were harvested and were subsequently plated and cultured in complete α-MEM medium containing M-CSF (30 ng/mL) and RANKL (50 ng/mL) for 5 days, with various concentrations of FABP4 (1000 ng/ml and 2000 ng/ml) (Tevlin et al. 2014; Zhang et al. 2024b). The culture medium was refreshed every other day until mature osteoclasts were formed. Following this, the cells were washed twice with PBS, fixed with 4% paraformaldehyde for 15 min, and stained for tartrate-resistant acid phosphatase (TRAP) activity. TRAP-positive cells with three or more nuclei were enumerated under a microscope. For each test, 3 Biological replicates were performed.

### ALP and ARS staining

mMSCs at the density of 1 × 10^5^/mL were seeded in a six-well plate. The cells were induced by OIM for 7 or 21 days, followed by ALP (ab242287, Abcam, Cambridge, MA, USA) or ARS staining (Sigma-Aldrich, St Louis, USA). The time point was selected based on previous publications, which suggest that ALP positivity was distinctly detectable by day 7, while the formation of mineralization nodules became markedly evident by day 21 of the osteogenic induction culture (Prins et al. 2024; Faure et al. 2025). Briefly, the cells were washed three times with PBS and then fixed with 4% paraformaldehyde for 10 min. ALP staining reagent or 0.2% ARS solution was added in each well for 30 min at 37 ℃. To quantify Alizarin Red staining, cells were stained with Alizarin Red S, and then the dye was extracted using acetic acid. The absorbance of the solubilized dye is measured at 405 nm using a microplate reader. After washing three times with PBS, the stained cells in each well were photographed.

### ALP activity assay

mMSCs were cultured with OIM for 7 days. Quantitative ALP measurements were accomplished as previously reported (Yin et al. 2019). Briefly, the ALP activity in the supernatant was measured using the Alkaline Phosphatase Assay Kit (Beyotime Biotech Inc., Jiangsu, China). After co-incubation of substrates and p-nitrophenol for 30 min at 37 ℃, the ALP activity was tested. Finally, the ALP activity was normalized to the total protein content measured by the bicinchoninic acid Protein Assay Kit (Beyotime Biotech Inc., Jiangsu, China).

### In vitro tube formation assay

For the tube formation assay in vitro, the 24-well plates were firstly coated with Matrigel (BD Biosciences, USA) and pre-incubated at 37 °C for 1 h. Next, EPCs at the density of 2.5 × 10^5^ cells were seeded in the well and cultured in Endothelial Growth Medium 2 (EGM2) for 10 h and then the number of branches was counted.

### Wound-healing assay

EPCs were seeded in a six-well plate and grown to nearly 100% confluency. The scratch assay was performed with a 200-μl pipette tip used to make a linear scratch and then the cells were washed with PBS twice. As the optimal concentration of FABP4 to EPCs has not yet been elucidated, low concentration (500 ng/ml) and high concentration (1000 ng/ml) of FABP4 were both selected. Images were taken at 0 h, 12 h, 24 h, and 48 h at 100 × magnification, and the wound size was measured in three wells per group (Chen et al. 2023).The number of photos used for quantification in the cell wound healing assay was six.

### Tube formation assay in vivo

EPCs were mixed with matrigel (BD Biosciences, USA) at a density of 1 × 10^5^/ml. The mixture of matrigel and EPCs was injected into the subcutaneous tissue of BALB/c nude mice (20–22 g, four weeks old). Nude mice were euthanized on the 7th day and then the matrigel was collected for the subsequent experiments (Buttler et al. 2014).

### qRT-PCR

qRT-PCR was performed on osteogenesis experiments. Total RNA was extracted from mMSCs using the Trizol reagent (Invitrogen, Paisley, UK). For reverse transcription of mRNA, first-strand cDNA was synthesized from 1 μg of total RNA using PrimeScript RT reagent kits: Cat#RR037A and Cat#RR036A (TaKaRa, Dalian, China). After reverse transcription reaction, real-time PCR was performed using a SYBR Green qRT-PCR kit (TaKaRa, Dalian, China) and an ABI Step One Plus Real-Time PCR System. β-actin was used as a reference for quantitation of mRNAs. The primer sequences used in this study were as follows, Osterix (accession no. AF184902.1): forward, 5'-GTCCTCTCTGCTTGAGGAAGAA-3'; reverse, 5'- GGGCTGAAAGGTCAGC GTAT-3'; Runx2 (accession no. NM_009820.5): forward, 5'-GTGTCACTGCGCTGAAGAGG-3'; reverse, 5'-G ACCAACCGAGTCATTTAAGGC-3'; ALP (accession no. X13409.1): forward, 5'-GGTAGATTACGCTCAC AACAA-3'; reverse, 5'-A GGCATACGCCATCACAT-3'; β-actin (accession no. NM_007393.4): forward, 5'-CTGTCCCTGTATGCCTCTG-3'; reverse, 5'-ATGTCACGCACGATTTCC-3'. TRAP: forward, 5'-CACTCCCACCCTGAGATTTGT-3'; reverse, 5'-CATCGTCTGCACGGTTCTG-3'.

### Western blot analysis

Western blot analysis was performed on osteogenesis and angiogenesis experiments. The protein samples were extracted from cells. The membrane was incubated overnight at 4 °C in one of the following primary antibodies: p-PI3K (ab278545, 1:500, Abcam, Cambridge, MA, USA), PI3K (ab154598, 1:500, Abcam, Cambridge, MA, USA), p-Akt (ab38449, 1:500, Abcam, Cambridge, MA, USA), Akt (ab300473, 1:500, Abcam, Cambridge, MA, USA), and GAPDH (ab8245, 1:500, Abcam, Cambridge, MA, USA) as an internal control. The membrane was incubated with a secondary antibody (ab288151, 1:5000, Abcam, Cambridge, MA, USA) for 2 h and detected using the Enhanced Chemiluminescence Western blot System (Amersham Biosciences).

### Label-free quantitative proteomics

The Label-free quantitative proteomics was performed as previously described (Chen et al. 2022). Briefly, knee subchondral bones from the mice fed with HFD and oral gavage with BMS309403 or PBS for 6 months were harvested. The samples were ground into powder in liquid nitrogen and underwent trypsin digestion. HPLC fractionation and LC–MS/MS analysis were performed subsequently. The resulting MS/MS data were retrieved using the MaxQuant search engine (v.1.5.2.8). The Bioinformatics analysis was performed. When the expression of a protein was 1.5 times higher or 2/3 lower in the experimental group (BMS309043-treated group) than in the control group (PBS-treated group) and the p-value was < 0.05, it was considered a differentially expressed protein (DEP). Gene Ontology (GO) annotated proteome was derived from the UniProt-GOA database (http://www.ebi.ac.uk/GOA/). The proteins were classified according to biological process (BP), cell component (CC), and molecular function (MF). Protein pathways were annotated using the Kyoto Encyclopedia of Genes and Genomes (KEGG) and Wikipathways database. ClueGO (V2.5.6) was further used for BP and KEGG enrichment analysis. In the parallel study, EPCs were cultured and FABP4 (1000 ng/ml) was added to stimulate EPCs for 24 h (Zhang et al. 2024b). The proteins were extracted and the Label-free quantitative proteomics was performed as described above. The interested DEPs were validated with parallel reaction monitoring (PRM).

### Statistical analysis

The composite data are expressed as means ± s.e.m. Statistical analysis was performed with one-way ANOVA followed by Dunnett’s test for multiple comparisons, otherwise student's t-test was used. Differences were considered to be significant at P ≤ 0.05. Analyses were performed in SPSS 23.0 software (IBM, USA).

## Results

### Knocking out of FABP4 significantly alleviated subchondral bone sclerosis and type H vessel formation in mice fed with HFD

**Fig. 1:**
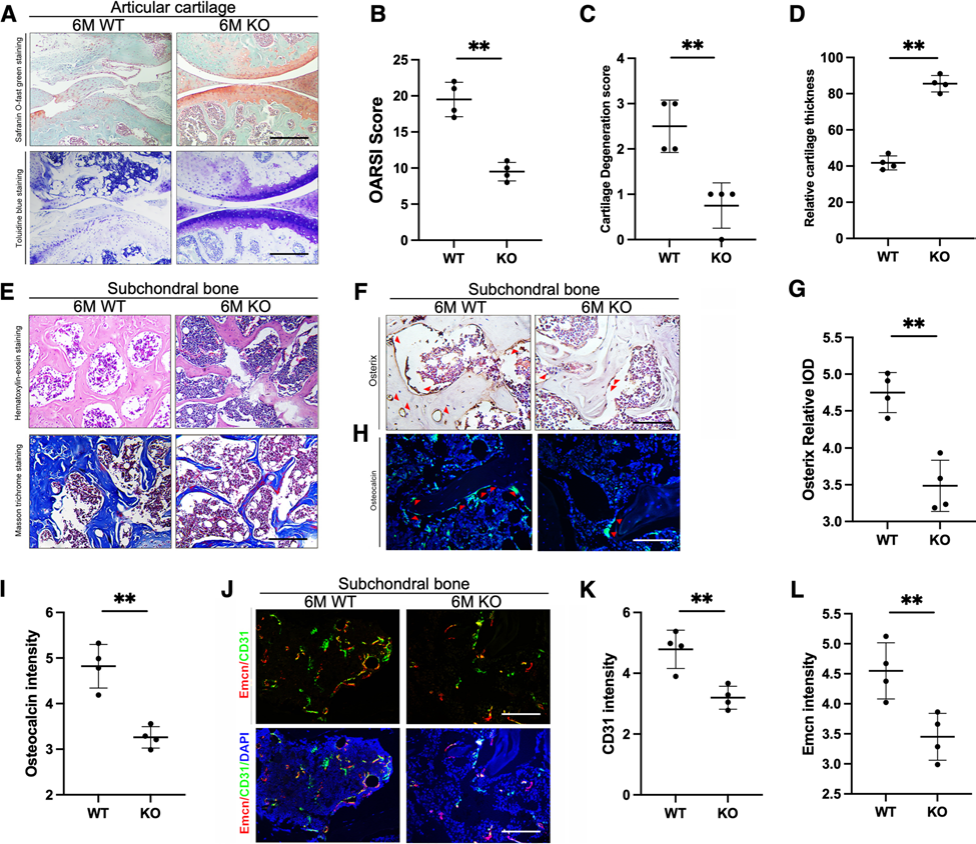
Knocking out of FABP4 significantly alleviated subchondral bone sclerosis and type H vessel formation in the mice fed with HFD for 6 months. **A** The representative images of Safranin O-fast green and Toluidine blue staining in the articular cartilage of the FABP4 WT mice and KO mice fed with 6-month HFD. (Scale bar = 300 μm) **B**–**D** OARSI score, cartilage degeneration score, and relative cartilage thickness were calculated between the FABP4 WT and KO mice. *(n* = 4, **P < *0.01).* **E** The representative images of hematoxylin–eosin (HE) and Masson trichrome staining showed the trabecular thickness and number in the subchondral bone between the FABP4 WT and KO mice. (Scale bar = 60 μm) **F** The representative images of immunohistochemistry images of osterix between the FABP4 WT and KO mice. (Scale bar = 60 μm) **G** The expression level of osterix (represented as relative integrated optical density (IOD)). (*n* = 4, **P < *0.01).* **H** The representative images of immunofluorescence staining of osteocalcin between the FABP4 WT and KO mice. Green: molecular probes marking osteocalcin, blue: DAPI marking nucleus. (Scale bar = 60 μm) **I** Statistical analyses of the relative fluorescence density of osteocalcin. (*n* = 4, **P < *0.01)* **J** The representative images of immunofluorescence staining of CD31 and Emcn between the FABP4 WT and KO mice. Green: molecular probes marking CD31, Red: molecular probes marking Emcn, blue: DAPI marking nucleus. (Scale bar = 60 μm) **K**–**L** Statistical analyses of the relative fluorescence intensity of CD31 (**K**) and Emcn (**L**). (*n* = 4, **P < *0.01).* (Student’s t-test was performed for the above analysis)

The Safranin O-fast green staining and Toluidine blue staining were first used to observe the degeneration of articular cartilage. Articular cartilage from the FABP4 WT mice fed with 6-month HFD suffered more severe destruction and loss of integrity, compared to the FABP4 KO mice (Fig. 1A). The OARSI score, cartilage degradation score, and relative cartilage thickness deteriorated in the WT mice with HFD, which were effectively improved in KO mice (Fig. 1B–D). HE staining and Masson trichrome staining were used to observe the change of subchondral bone in FABP4 KO and WT mice. The KO mice had significantly alleviated sclerosis than the WT mice after 6 months of HFD (Fig. 1E). The expressions of osteogenic markers osterix and osteocalcin (OCN) in subchondral bone were detected separately by immunohistochemistry (Fig. 1F) and immunofluorescence (Fig. 1H). The results showed that the number of osterix-positive cells with brown staining was reduced in the subchondral bone of KO mice compared with WT mice after 6 months of HFD (Fig. 1G) and similarly, the green fluorescence intensity of OCN was weakened in KO mice (Fig. 1I).

In addition to increasing bone formation in subchondral bone, significant promoting type H vessel formation, presented with specific cell surface markers CD31 and endomucin (CD31hi Emcnhi), was noted in the WT mice after HFD. However, the knock-out of FABP4 alleviated the promoting effect of type H vessel formation in mice fed with HFD (Fig. 1J), with CD31 and Emcn intensity significantly lower in these groups (Fig. 1 K, L). This data suggests knocking out of FABP4 in mice fed with HFD significantly alleviated subchondral bone type H vessel formation.

Similar results were found in mice fed with 3 months of HFD. (Supplemental Fig. 1). While there were no significant differences in mice treated with ND either after 3 months or 6 months. (Supplemental Fig. 2).

**Fig. 2:**
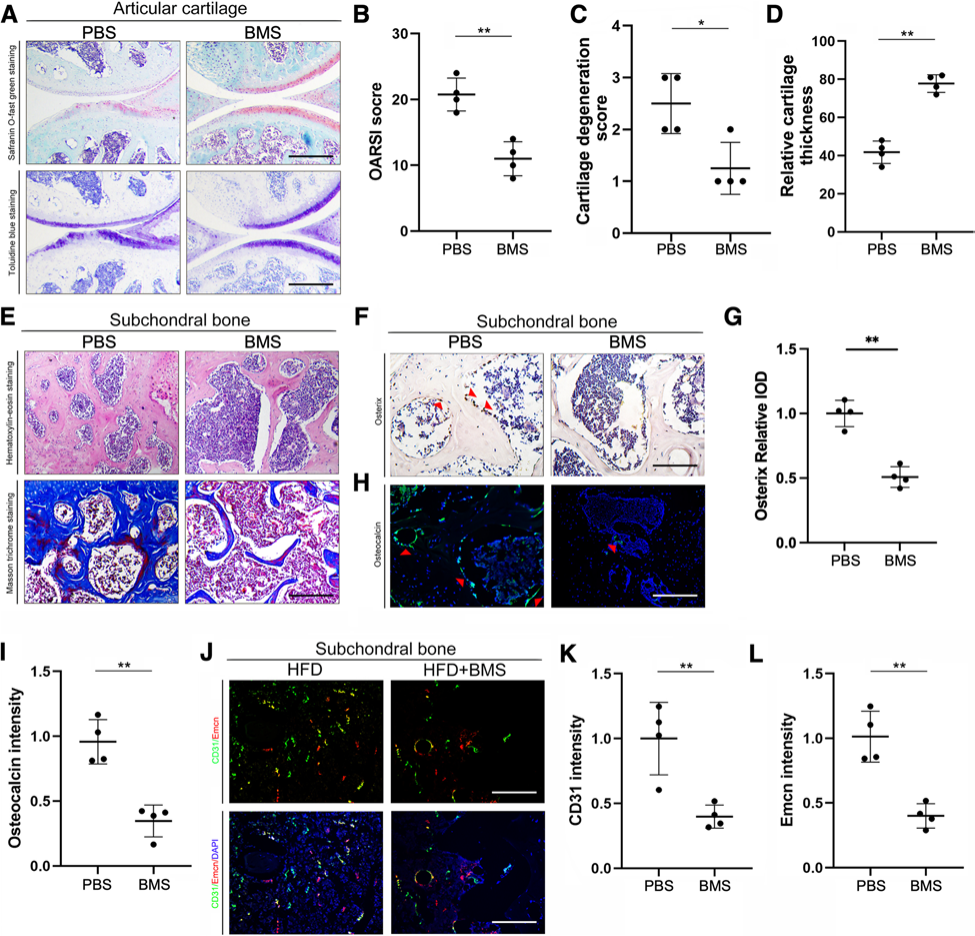
Pharmaceutical inhibition of FABP4 significantly alleviated subchondral bone sclerosis and type H vessel formation in mice fed with HFD for 6 months. **A** The representative images of Safranin O-fast green and Toluidine blue staining in the articular cartilage of HFD-fed mice with PBS or BMS309403. (Scale bar = 300 μm) **B**–**D** OARSI score, cartilage degeneration score, and relative cartilage thickness were calculated between PBS and BMS309403. *(n* = 4, *P < 0.05, **P < *0.01)* **E** The representative images of HE and Masson trichrome staining showed the trabecular thickness and number in the subchondral bone. (Scale bar = 60 μm) **F** The representative images of immunohistochemistry images of osterix. (Scale bar = 60 μm) **G** The expression level of osterix (represented as relative IOD). (*n* = 4, **P < *0.01)* **H** The representative images of immunofluorescence staining of osteocalcin. Green: molecular probes marking osteocalcin, blue: DAPI marking nucleus. (Scale bar = 60 μm) **I** Statistical analyses of the relative fluorescence density of osteocalcin. (*n* = 4, **P < *0.01)* **J** The representative images of immunofluorescence staining of CD31 and Emcn. Green: molecular probes marking CD31, Red: molecular probes marking Emcn, blue: DAPI marking nucleus. (Scale bar = 60 μm) **K**–**L** Statistical analyses of the relative fluorescence density of CD31 (**K**) and Emcn (**L**). (*n* = 4, **P < *0.01)* (Student’s t-test was performed)

### Pharmaceutical inhibition of FABP4 significantly alleviated subchondral bone sclerosis and type H vessel formation in mice fed with HFD

To investigate the effects of inhibiting FABP4 on the subchondral bone, WT mice were fed with BMS309403 with oral gavage for 6 months. Articular cartilage from the mice fed with HFD suffered more severe destruction and loss of integrity, which were alleviated by BMS309403 (Fig. 2A). The OARSI score, cartilage degradation score, and relative cartilage thickness deteriorated in the mice with HFD feeding, and were effectively improved by BMS309403 (Fig. 2B–D). The HE staining and Masson trichrome staining showed that the subchondral bone sclerosis in the BMS group was decreased as compared with that in the PBS group (Fig. 2E). Furthermore, the results of immunohistochemistry and immunofluorescence showed that expressions of osterix and osteocalcin were decreased in the BMS group compared with the PBS group (Fig. 2F–I). For the formation of type H vessels, the results showed that CD31hi Emcnhi vessels were reduced in the subchondral bone of mice with BMS (Fig. 2J-L).

### Knocking out of FABP4 significantly alleviated subchondral bone sclerosis

**Fig. 3:**
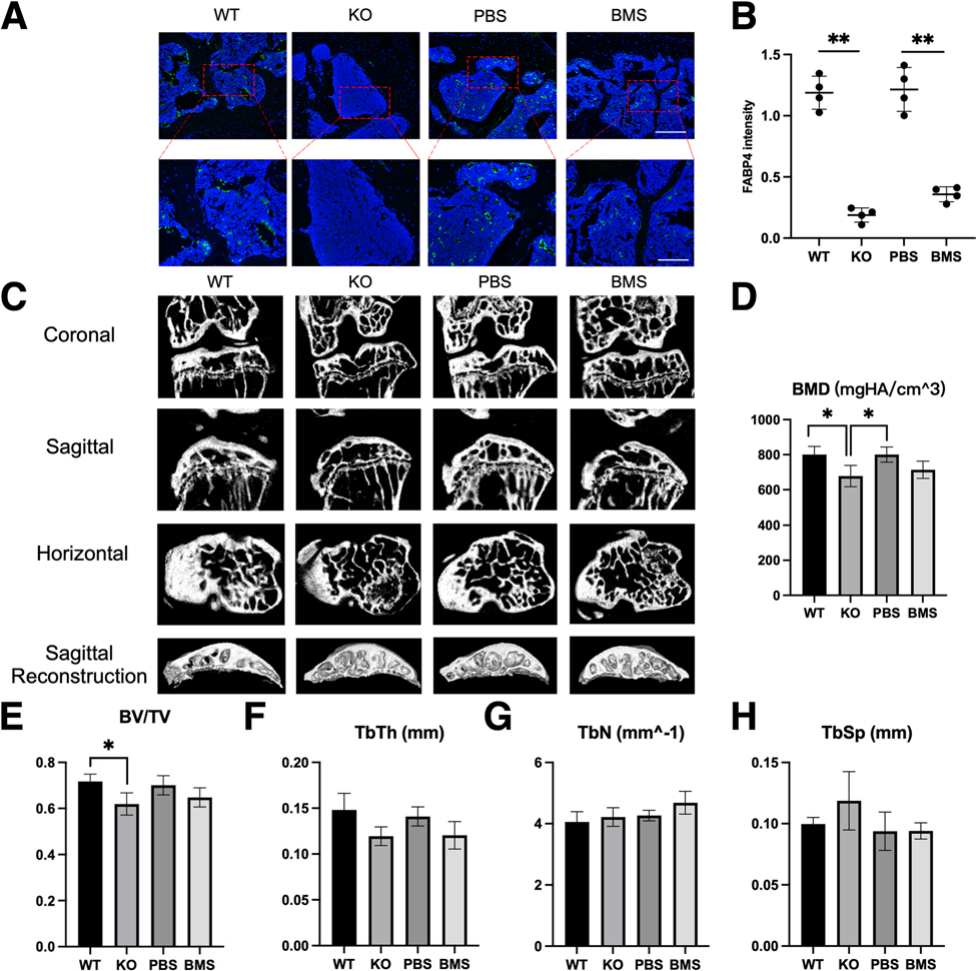
Knocking out of FABP4 significantly alleviated subchondral bone sclerosis in the mouse fed with HFD. **A** The representative images of FABP4 in the subchondral bone of FABP4 WT mice, FABP4 KO mice, mice fed with PBS, and mice fed with BMS309403. (Scale bar = 60 μm) **B** The expression level of FABP4 (represented as FABP4 intensity). (*n* = 4, **P < *0.01).* **C** Three-dimensional CT scans of knee joints in the WT, KO, PBS, and BMS groups. **D–H** Histogram showing the statistical analysis of BMD, BT/TV, TbTh, TbN, and TbSp among the WT, KO, PBS, and BMS groups. (*n* = 4, *P < *0.05)* (One-way ANOVA followed by Dunnett’s test was performed)

The three-dimensional micro-CT images of the knee in WT and KO mice, and in mice treated with PBS and BMS were performed. The KO mice showed no staining for FABP4 and the mice treated with BMS309403 showed decreased staining for FABP4 (Fig. 3A, B). The micro-CT figures demonstrated severe subchondral bone sclerosis in WT mice after 6 months of HFD, compared with the KO mice (Fig. 3C). The mice fed with PBS had seemingly more severe subchondral bone sclerosis than the mice fed with BMS309403 (Fig. 3C).

The 3D analysis of subchondral bone showed significantly higher BMD and BV/TV of WT mice than the KO mice (Fig. 3D, E). However, there were no statistical differences in Th. Th, Tb. N, and Tb.Sp between the 2 groups (Fig. 3F, G, H). Meanwhile, treatment of BMS309403 (30 mg/kg/d) for 6 months did not have statistically significant effects on the subchondral bone (Fig. 3D–H). Additionally, we also performed a proteinomics study and found that inhibition of FABP4 was significantly associated with the biological processes including bone remodeling and angiogenesis and the PI3K-Akt-mTOR signaling pathway may be a crucial pathway during the process. (Supplemental Fig. 3).

Taken together, these results suggested that knocking out of FABP4 and pharmaceutical inhibition of FABP4 alleviated subchondral bone sclerosis and type H vessel formation in the knee joints of mice fed with HFD.

### FABP4 promotes the differentiation of MSCs into osteoblasts through activation of the PI3K/Akt signaling pathway in vitro

**Fig. 4:**
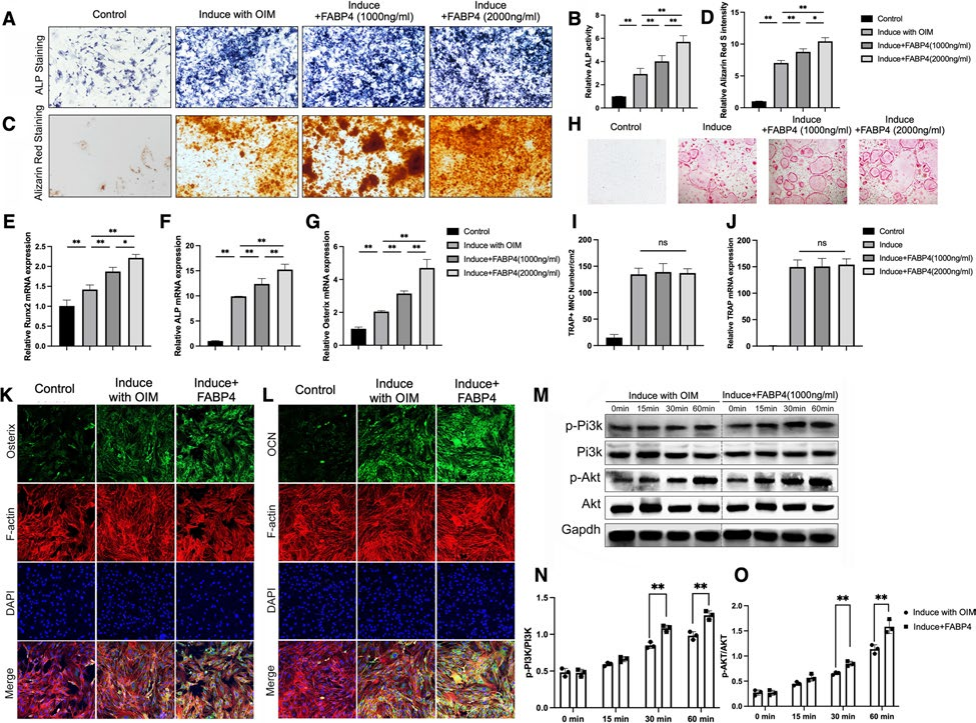
FABP4 promotes the differentiation of BMSCs into osteoblasts through activation of the PI3K/Akt signaling pathway. **A** The representative images of ALP staining in BMSCs treated with FABP4 at low concentration (1000 ng/ml) or high concentration (2000 ng/ml) under OIM for 7 days. **B** ALP activity assay was accomplished in BMSCs treated with FABP4 for 7 days. (*n* = 3, **P < *0.01)* **C** Representative ARS staining images in BMSCs treated with FABP4 (1000 ng/ml or 2000 ng/ml) for 21 days. **D** Quantitative analyses of ARS staining. (*n* = 3, *P < 0.05, **P < *0.01)* **E**–**G** qRT-PCR analysis and quantitation of Runx2 (**E**), ALP (**F**), and Osterix (**G**) expressions in BMSCs treated with FABP4 (1000 ng/ml or 2000 ng/ml) for 21 days. (*n* = 3, *P < 0.05, **P < *0.01)* **H** The representative images of TRAP staining in BMMs treated with FABP4 at low concentration (1000 ng/ml) or high concentration (2000 ng/ml). **I–J** TRAP + number **I** and relative TRAP mRNA expression level (I). (*n* = *3)* **K–L** Representative immunofluorescence staining of osterix (K) and OCN **L** in BMSCs treated with FABP4 (1000 ng/ml) for 5 days. Green: molecular probes marking osteocalcin and osterix, blue: DAPI marking nucleus. **M**–**O** Western blot and quantitation of PI3K/Akt signaling and corresponding statistical analysis of p-PI3K/PI3K **N** and p-Akt/Akt **O** expression in BMSCs treated with or without FABP4 (1000 ng/ml) for 15 min, 30 min, and 60 min. (*n* = 3, **P < *0.01)* (One-way ANOVA followed by Dunnett’s test was performed)

To determine the effect of FABP4 on bone formation, we first used ALP staining, ARS staining, and ALP activity assay to monitor osteoblast differentiation. We found that both 1000 ng/ml and 2000 ng/ml of FABP4 enhanced ALP staining and ALP activity at day 7 significantly (Fig. 4A, B). Furthermore, ARS red staining showed increased mineral deposition in BMSCs treated with FABP4 for 21 days (Fig. 4C, D). In addition, compared with the control group, the treatment of FABP4 significantly increased expressions of the osteogenesis-related markers of runt-related transcription factor 2 (Runx2), ALP, and Osterix, as revealed by qRT-PCR (Fig. 4E–G). To further investigate the effect of FABP4 on osteoclast formation in vitro, BMMs were induced in the presence or absence of FABP4. TRAP staining revealed that the number of mature osteoclasts was not significantly affected by FABP4 at either concentration. (Fig. 4H) Consistent with these findings, FABP4 did not inhibit TRAP gene expression (Fig. 4I–J). Consistent with the results of qRT-PCR for osteogenesis-related markers, the results of immunofluorescence showed that the green fluorescence intensity of osteogenesis-related markers, Osterix and OCN, were higher in the FABP4 group than the control group (Fig. 4K, L). These results indicated that FABP4 promotes the differentiation of MSCs into osteoblasts in vitro.

To gain insights into the molecular mechanisms by which FABP4 promotes osteoblast differentiation, as revealed by the enrichment analysis, we tested the role of PI3K/AKT signaling pathways, one critical signaling pathway for bone formation, during the process of FABP4-increased osteoblasts differentiation. Western blotting results demonstrated that phosphorylation of PI3K and Akt, which were increased by OIM and were further increased by FABP4 treatment (Fig. 4M–O). These results suggested that FABP4 promoted the differentiation of MSCs into osteoblasts through activation of the PI3K/Akt signaling pathway.

### FABP4 promotes endothelial tube formation and wound healing through activation of the PI3K/Akt signaling pathway in vitro

**Fig. 5:**
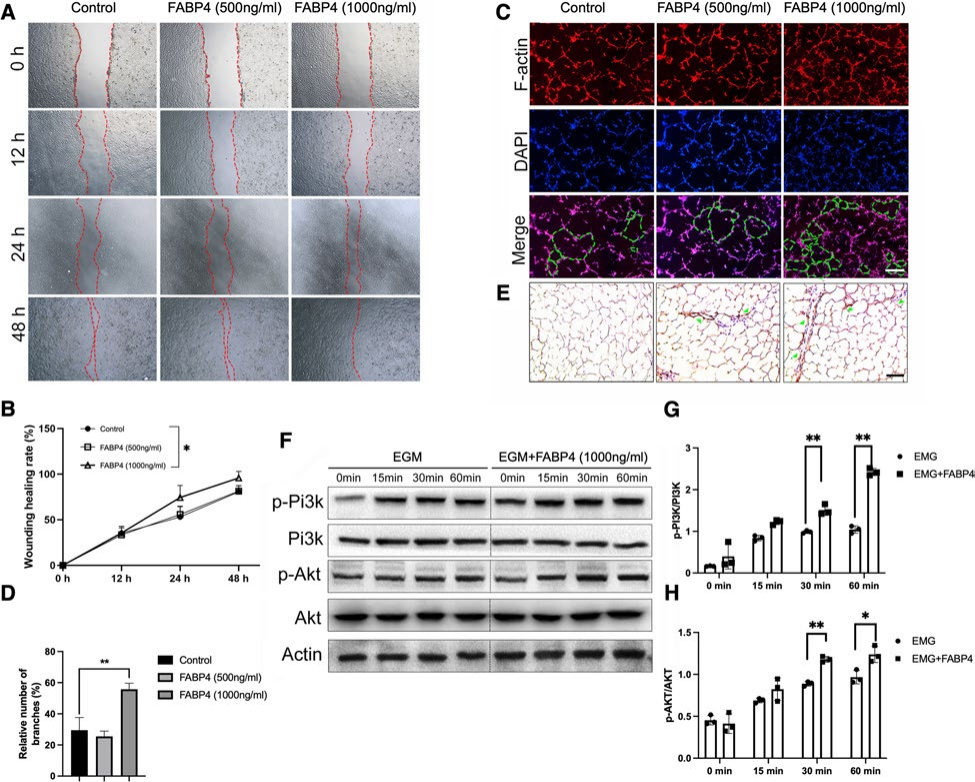
Promotion of endothelial cell migration, tube formation, and wound healing by FABP4 in vitro*.* **A** The scratch assay of EPCs treated with FABP4 at low concentration (500 ng/ml) or high concentration (1000 ng/ml) for 12 h, 24 h, and 48 h, observed by a scanning electron microscope. **B** The wounding healing rate of EPCs summarized from the scratch assay. (*n* = 3, *P < *0.05).* **C**–**D** The representative images of Matrigel tube formation assay **C** and histogram showing quantitative analysis of the number of branches **D** in EPCs treated with FABP4 at 500 ng/ml or 1000 ng/ml for 10 h. (*n* = 3, **P < *0.01,* Scale bar = 75 μm) **E** The representative images of HE staining of the number of tube formations in vivo. (Scale bar = 75 μm) **F** Western blot analysis and quantitation of PI3K/Akt signaling and corresponding statistical analysis of p-PI3K/PI3K **G** and p-Akt/Akt **H** expression in EPCs treated with or without FABP4 (1000 ng/ml) for 15 min, 30 min, and 60 min. (*n* = 3, **P < *0.01).* (One-way ANOVA followed by Dunnett’s test was performed)

To determine whether FABP4 affected the angiogenesis of EPCs, tube formation and wound healing were assayed. Wound healing assays revealed that FABP4 (1000 ng/ml) promoted the migration of EPCs (Fig. 5A, B). Additionally, analysis of tube formation in EPCs showed that FABP4 promoted the angiogenesis process of EPCs with enhanced tube length and a higher number of intersections (Fig. 5C, D). Similar results were further examined by tube formation assay in vivo. HE staining showed that the number of tube formations (dark brown staining) was increased in matrigel mixed with FABP4-treated EPCs (Fig. 5E). To further investigate the molecular mechanisms by which FABP4 promotes angiogenesis, as revealed by the enrichment analysis, we tested the role of PI3K/AKT signaling pathways during the process of FABP4-increased angiogenesis. Western blotting results demonstrated that phosphorylation of PI3K and Akt, which were increased by EGM and were further increased by FABP4 (1000 ng/ml) treatment (Fig. 5F–H). These results suggested that FABP4 promoted EPCs through activation of the PI3K/Akt signaling pathway. Additionally, we also performed a proteinomics study and found that FABP4 regulated the expression of osteoblastic and osteoclastic markers in EPCs. The parallel reaction monitoring (PRM) analysis demonstrated that osteoblastic markers in EPCs (Thy1, Alpl, Lpl, Il1rn, Cdh1l, Adamts 1, Il6st, Col6a2, Col6a1, Postn, and Itch) were upregulated after the stimulation of FABP4 (Supplemental Fig. 4).

## Discussion

The pathogenesis of OA is not yet fully understood. Our previous studies have shown that a novel adipokine, FABP4, plays an important role in the pathogenesis of OA (Zhang et al. 2018a, 2018b, 2024a). Mao et al. also demonstrated that FABP4 knockdown could markedly delay inflammation, apoptosis, and extracellular matrix degradation in chondrocytes in vitro (Mao et al. 2021). However, the effects of FABP4 on subchondral bone remodeling and angiogenesis, and whether it plays a role in the pathogenesis of metabolic OA have not been reported.

In this study, an HFD-induced obesity animal model was used to evaluate the role of FABP4 in metabolic OA, as previous studies have demonstrated the effects of HFD in inducing OA (Griffin et al. 2010, 2012; Barboza et al. 2017; Donovan et al. 2018). Consistent with previous studies (Zhang et al. 2018a), the present results demonstrate that HFD can significantly induce obesity in mice. As a result, OA-like pathological changes were widely seen in these groups of mice (cartilage degenerative changes and subchondral bone sclerosis), while knocking out or pharmaceutical inhibition of FABP4 could significantly alleviate cartilage degradation. Meanwhile, knockout of FABP4 markedly alleviated subchondral bone sclerosis and reduced the expression of osteogenic markers including Osterix and Osteocalcin. Moreover, knockout of FABP4 significantly decreased angiogenesis and type H vessel formation in the subchondral bone. Proteomic enrichment analysis revealed that inhibiting FABP4 led to significant alterations in bone remodeling, angiogenesis, and inflammation. Additionally, the FABP4-specific inhibitor BMS304630 could markedly delay subchondral bone sclerosis and angiogenesis. In vitro studies further confirmed that FABP4 could activate the PI3K/AKT pathway to induce osteoblast differentiation and osteogenesis, as well as angiogenesis and endothelial cell migration through the same pathway. Furthermore, we verified that FABP4 could induce the expression of osteogenic markers (including Alpl, Lpl, Il6st, Col6a1, Col6a2, etc.) in endothelial progenitor cells. In summary, these results demonstrate that FABP4 can directly induce osteoblast differentiation and subchondral bone sclerosis. Meanwhile, FABP4 can also facilitate angiogenesis and osteogenic differentiation by inducing endothelial cell differentiation and type H vessel formation. The PI3K/AKT pathway plays an important role in both processes.

The role of FABP4 in bone remodeling remains unclear. Results from Guo et al. also showed that FABP4 was significantly positively correlated with osteoclast numbers (Guo et al. 2022). However, these were in vitro findings not verified in OA human or animal models. In contrast, through in vitro and in vivo experiments, our study verified that in a metabolic OA model, FABP4 can promote osteoblast proliferation and differentiation to facilitate osteogenesis. This process was mainly achieved by activating the PI3K/AKT signaling pathway. Subsequently, the promotion of subchondral bone formation exacerbated subchondral bone sclerosis, contributing to OA (Donell 2019).

Additionally, extensive angiogenesis in subchondral bone has been widely reported in the literature (Liu et al. 2023). In many previous studies, adipokines have been shown to play important roles in promoting angiogenesis. Leptin has been identified as a potent angiogenic factor in the cardiovascular system (Khazaei and Tahergorabi 2015). Adiponectin has also been found to promote angiogenesis in neonatal mice (Shah et al. 2022). While for FABP4, several studies outside the field of OA have investigated its pro-angiogenic effects. FABP4 promotes angiogenesis in tumors, and in vivo, the application of FABP4 siRNA exhibited anti-angiogenic and anti-tumor effects (Harjes et al. 2017). In endothelial cells, FABP4 also promotes angiogenesis (Elmasri et al. 2012; Harjes et al. 2014). In a recent study, Guo et al. found that in RA models, FABP4 could induce proliferation and differentiation of vascular endothelial cells (Guo et al. 2022). Our study, both in vivo and in vitro, found that similar to other adipokines, FABP4 also has a role in promoting angiogenesis in metabolic OA models. Likewise, this was achieved by activating the PI3K/AKT pathway, analogous to osteogenesis promotion. This may be another critical factor facilitating OA occurrence. Recent studies have shown that H-type vessels are a specialized vessel subtype accompanying bone formation and are important for osteogenesis.

Increased H-type vessels have also been observed in OA pathogenesis (Liu et al. 2023). Recent studies have shown increased expression of angiogenic growth factors in articular cartilage during OA development (Shao et al. 2015). Angiogenesis during OA can impact chondrocyte proliferation and apoptosis, accelerate extracellular matrix mineralization, promote osteophyte formation, and exacerbate subchondral bone remodeling, further contributing to cartilage degeneration, altered mechanical loading, and OA progression (Lampert et al. 2016). Additionally, angiogenesis can regulate neuro-related factors and induce nerve ingrowth, eventually leading to joint pain in OA patients (Yang et al. 2011). Moreover, angiogenesis facilitates macrophage infiltration and production of various inflammatory factors, affecting cell self-repair (Zhu et al. 2007). Consistent with previous studies, our research found significantly increased H-type vessels in OA subchondral bone. More importantly, angiogenesis could also promote the expression of osteogenic proteins including but not limited to Alpl, Lpl, Il6st, Col6a1, and Col6a2, facilitating subchondral sclerosis. FABP4 plays a key role in this process. Inhibition of FABP4 may serve as a method to suppress H-type vessel regeneration and a potential OA treatment approach.

We previously analyzed the signaling pathways of adipokines in OA (Zhang et al. 2022). This study also demonstrated that the PI3K-Akt signaling pathway plays a crucial role in FABP4-induced osteogenesis and angiogenesis. The PI3K-Akt signaling pathway functions in many cellular processes that are essential for homeostasis, including the cell cycle, survival, metabolism, inflammation, and apoptosis (Tang et al. 2018). Molecules, such as adipokines and cytokines, can activate receptor tyrosine kinases and G protein-coupled receptors and then activate PI3K to generate phospholipase (Santis et al. 2019). Akt, as a downstream effector of PI3K, is subsequently activated by these signals. Activated Akt is transferred to other cellular compartments to activate various downstream substrates, including metabolic enzymes, protein kinases, small G protein regulators, E3 ubiquitin ligases, and transcription factors (Manning and Toker 2017). The PI3K-Akt signaling pathway plays an important regulatory role in cartilage homeostasis, subchondral bone dysfunction, and synovitis (Sun et al. 2020). Several studies have been performed to show adipokines play important roles in the pathogenesis of OA via the PI3K Akt pathway (Zhang et al. 2022). Our study, for the first time, demonstrated that FABP4 induces osteoblastogenesis and angiogenesis via the pathway. Targeting the PI3K-Akt pathway may also serve as an OA treatment approach, warranting further investigation in future studies.

This study also has several limitations. First, the optimal treatment strategy of BMS309403 was not investigated in this study, as the intervention methods for BMS309403 vary across studies, using different models: Feng et al. used 50 mg/kg/day by gavage for 7 days (Feng et al. 2020), while Yao used 40 mg/kg/day by gavage for 4 weeks (Yao and JIANG DD et al. 2021). Others used 20 mg/kg injected locally every 3 days for 4 times (Lee et al. 2019). Second, this study used an HFD-induced animal model to monitor metabolic OA, while for the aging-related or surgery-related OA model, the role of FABP4 warrants future investigation for confirmation.

## Conclusions

This study demonstrates that FABP4 plays a pivotal role in the pathogenesis of metabolic OA. Specifically, FABP4 promotes subchondral bone osteogenesis and angiogenesis, potentially through activation of the PI3K-Akt signaling pathway. These findings suggest that targeting FABP4 inhibition could represent a promising therapeutic strategy for the treatment of metabolic OA.

## Electronic supplementary material

Below is the link to the electronic supplementary material.

Supplementary Material 1.

## Data Availability

No datasets were generated or analysed during the current study.

## Abbreviations

FABP4: Fatty acid binding protein 4
OA: Osteoarthritis
KO: Knockout
WT: Wild-type
HFD: High-fat diet
mMSCs: Mouse mesenchymal stem cells
EPCs: Endothelial progenitor cells
PI3K: Phosphatidylinositol-3-Kinase
ALP: Alkaline phosphatase
OIM: Osteoblastic induction medium
EGM: Endothelial growth medium
PRM: Parallel reaction monitoring
DEP: Differentially expressed protein
GO: Gene ontology
BP: Biological process
CC: Cell component
MF: Molecular function
KEGG: Kyoto encyclopedia of genes and genomes
CT: Computed tomography
ROI: Region of interest
BMD: Bone mineral density
BV/TV: Bone volume percentage
Tb. Th: Trabecular thickness
Tb. N: Trabecular number
Tb. Sp: Trabecular separation
DAPI: 4',6-Diamidino-2-phenylindole
VEGF: Vascular endothelial growth factor
qRT-PCR: Quantitative reverse transcription polymerase chain reaction
ANOVA: Analysis of variance
H&L: Heavy and light chains
IgG: Immunoglobulin G
PBS: Phosphate-buffered saline
OPG: Osteoprotegerin
mTOR: Mechanistic target of rapamycin
NF-κB: Nuclear factor kappa B
