# Supplementary material for: Fatty acid binding protein 4 induces osteogenesis and angiogenesis as pathogenesis of metabolic osteoarthritis
Source: Mol Med. 2025 Dec 19;32:9. doi: 10.1186/s10020-025-01330-2 (PMC12831317; doi:10.1186/s10020-025-01330-2)
Supplement: Supplementary file 1 — Supplementary Material 1. [file 10020_2025_1330_MOESM1_ESM.docx]

**Supplementary Materials**

**
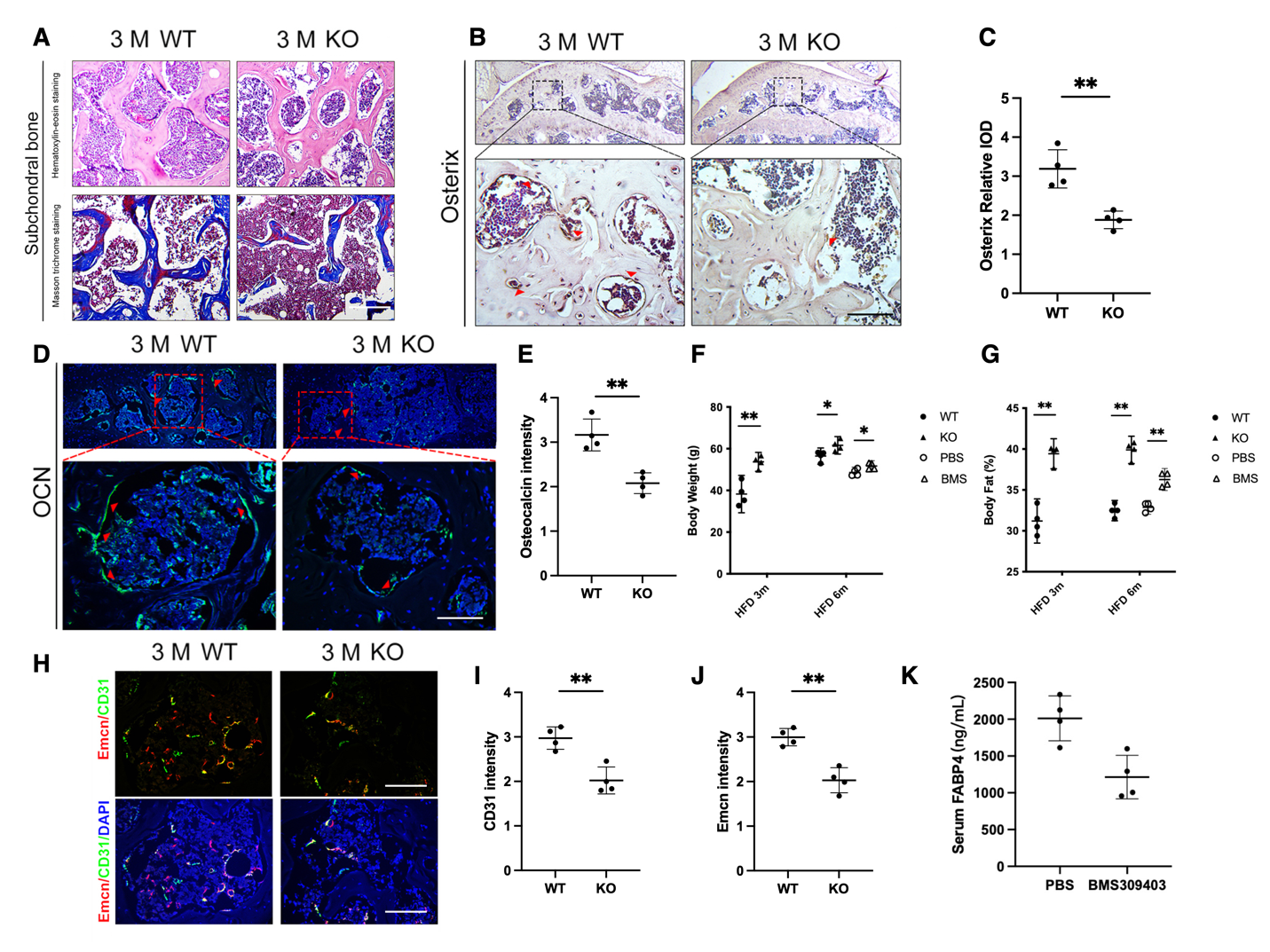
Supplemental Figure 1. Knocking out of FABP4 significantly alleviated subchondral bone sclerosis and type H vessel formation in the mice fed with 3-month HFD.**

(A) The representative images of hematoxylin-eosin (HE) and Masson trichrome staining showed the trabecular thickness and number in the subchondral bone between FABP4 WT and KO mice after 3-month HFD. (Scale bar = 60 μm) (B) The representative images of immunohistochemistry images of osterix between FABP4 WT and KO mice after 3-month HFD. (Scale bar = 60 μm) (C) The expression level of osterix (represented as relative integrated optical density (IOD)). (*n = 4, **P < 0.01).* (D) The representative images of immunofluorescence staining of osteocalcin between FABP4 WT and KO mice after 3-month HFD. Green: molecular probes marking osteocalcin, blue: DAPI marking nucleus. (Scale bar = 60 μm) (E) Statistical analyses of the relative fluorescence density of osteocalcin. (*n = 4, **P < 0.01).* (F) Body weight of WT or KO mice, and mice fed with PBS or BMS309403. *(n = 4, **P < 0.01, *P < 0.05).* (G) Body fat percentage of WT or KO mice, and mice fed with PBS or BMS309403. *(n = 4, **P < 0.01).* (H) The representative images of immunofluorescence staining of CD31 and Emcn between FABP4 WT and KO mice after 3-month HFD. Green: molecular probes marking CD31, Red: molecular probes marking Emcn, blue: DAPI marking nucleus. (Scale bar = 60 μm) (I-J) Statistical analyses of the relative fluorescence density of CD31 (I) and Emcn (J). (*n = 4, **P < 0.01).* (K) Serum FABP4 concentration of mice fed with HFD and PBS or BMS309403 for 6 months. (Student's t-test was performed for the above analysis)

**
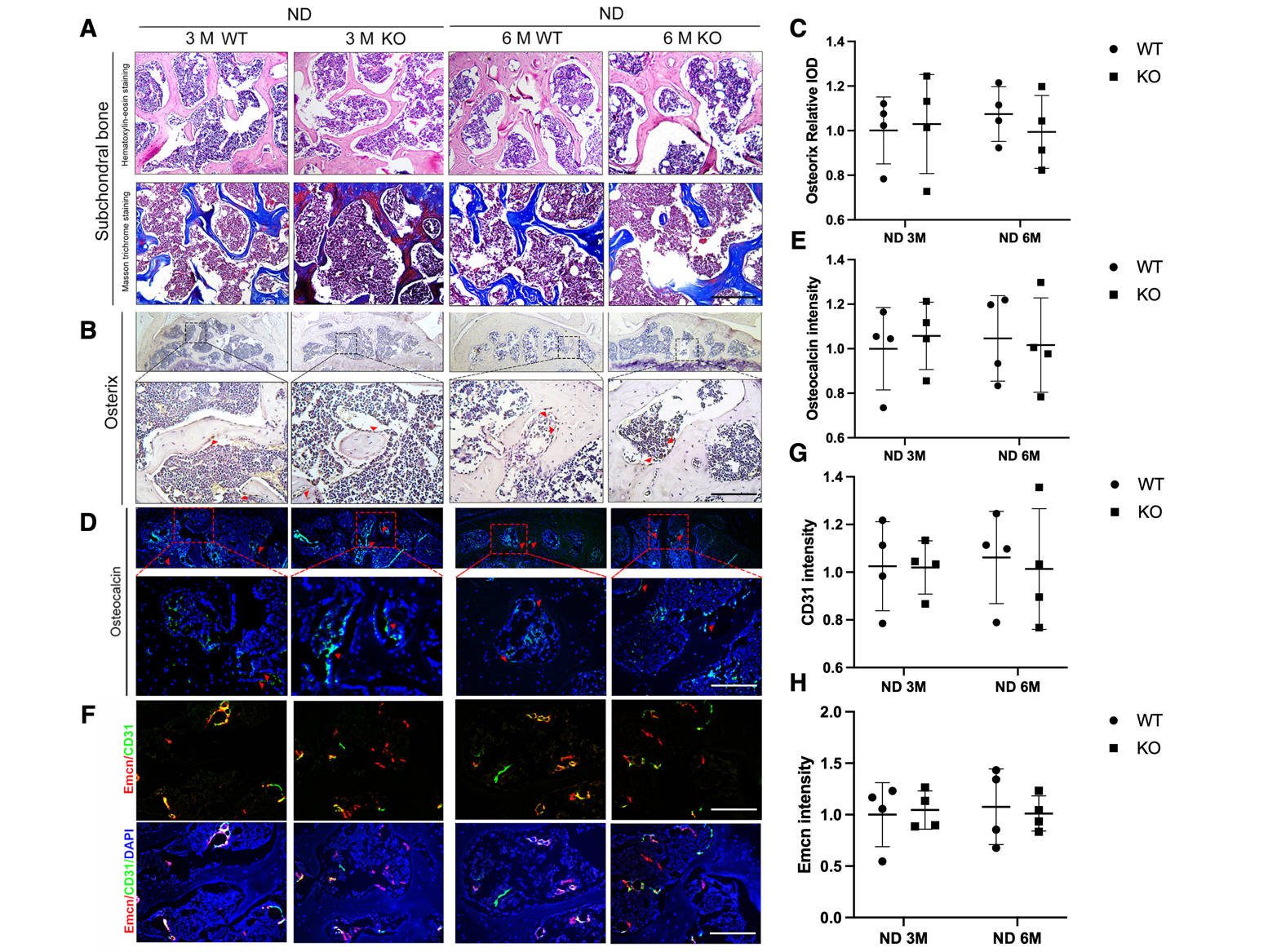
**

**Supplemental Figure 2. Knocking out of FABP4 has no effects on subchondral bone sclerosis and type H vessel formation in the mice fed with ND.**

(A) The representative images of HE and Masson trichrome staining in the subchondral bone between the FABP4 WT and KO mice after 3-month or 6-month ND. (Scale bar = 60 μm) (B) The representative images of immunohistochemistry images of osterix between the FABP4 WT and KO mice after 3-month or 6-month ND. (Scale bar = 60 μm) (C) The expression level of osterix. (*n = 4)* (D) The representative images of immunofluorescence staining of osteocalcin between the FABP4 WT and KO mice after 3-month or 6-month ND. Green: molecular probes marking osteocalcin, blue: DAPI marking nucleus. (Scale bar = 60 μm) (E) Statistical analyses of the relative fluorescence density of osteocalcin. (*n = 4)* (F) The representative images of immunofluorescence staining of CD31 and Emcn between the FABP4 WT and KO mice after 3-month or 6-month ND. Green: molecular probes marking CD31, Red: molecular probes marking Emcn, blue: DAPI marking nucleus. (Scale bar = 60 μm) (G-H) Statistical analyses of the relative fluorescence density of CD31 (G) and Emcn (H). (*n = 4)* (Student's t-test was performed for the above analysis)

***Inhibition of FABP4 was significantly associated with the biological processes including bone remodeling and angiogenesis.***

Label-free quantitative proteomics was used to obtain information on the protein changes in subchondral bone. A total of 4,487 proteins were identified, of which 3,490 were quantifiable.

Ultimately, 32 upregulated proteins and 25 downregulated proteins were detected (Supplemental Figure 3A). Among these DEPs, as expected, osteoblastic markers (Alpl, Edil3, Enpp1, and Olfml3) and angiogenic markers (Ang and Itgav) were downregulated, while osteoclastic markers (Hpgds) were upregulated (Supplemental Figure 3A). The GO enrichment analysis was performed on these 57 DEPs, including annotation of biological processes, cell components, and molecular function. The data showed that these DEPs were involved in biological processes including ossification, bone remodeling, bone growth, angiogenesis, and blood vessel development (Supplemental Figure 3B). They were mainly derived from the extracellular matrix (Supplemental Figure 3C) and had molecular functions such as glycosaminoglycan binding (Supplemental Figure 3D). The KEGG enrichment analysis showed that the DEPs were significantly enriched in the osteoclast differentiation and the PI3K-Akt signaling pathway (Supplemental Figure 3E). The WikiPathway enrichment analysis showed that the DEPs were significantly enriched in osteoclast signaling, endochondral ossification, the PI3K-Akt-mTOR signaling pathway, and the IL-3 signaling pathway (Supplemental Figure 3F).

**
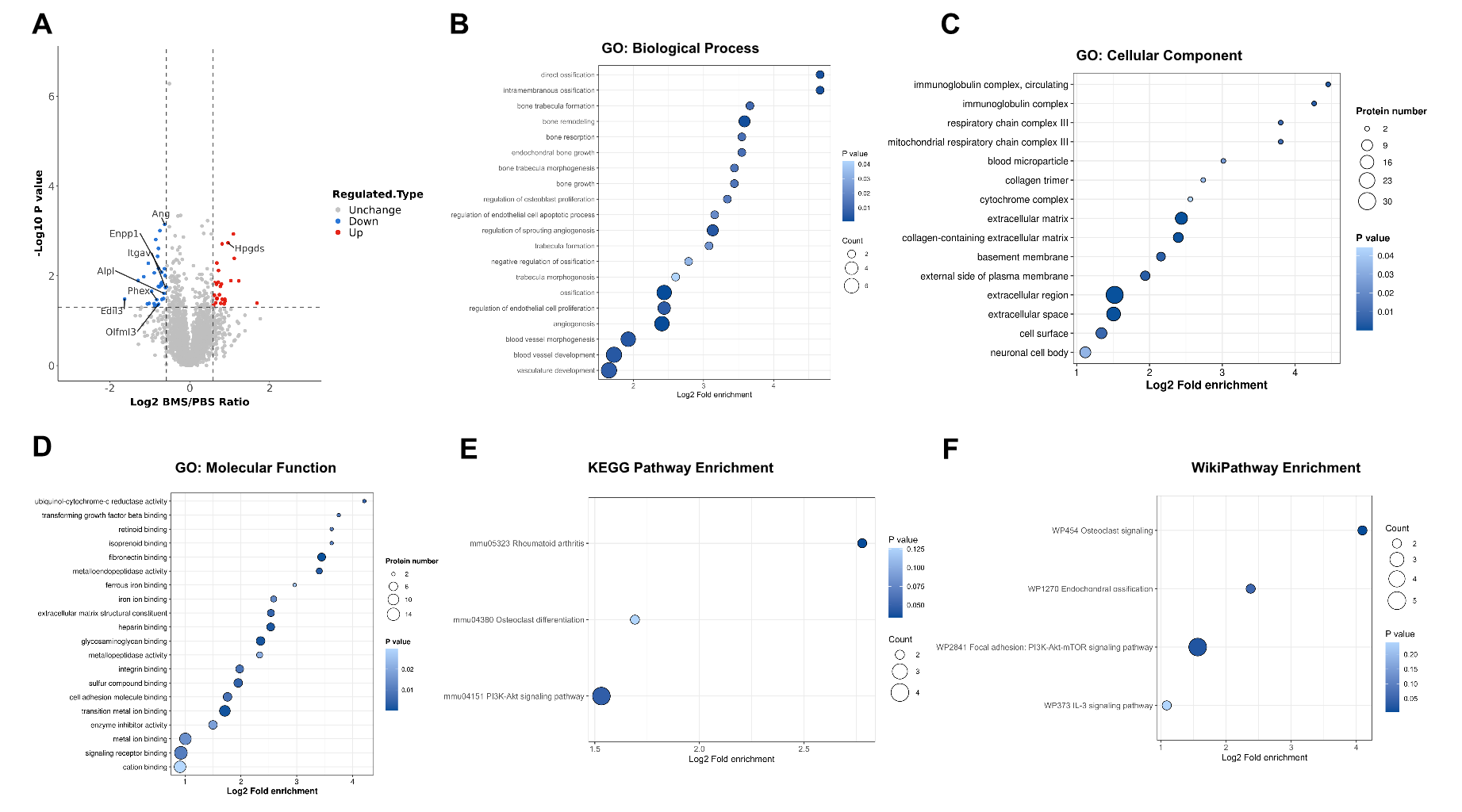
**

**Supplemental Figure 3. Inhibiting FABP4 was significantly associated with the biological processes including bone remodeling and angiogenesis.** (A) The volcano plot illustrated the number of proteins quantified. The blue spots on the left are proteins that are down-regulated 1.5-fold in the BMS309403 group and the red spots on the right are proteins that are up-regulated 1.5-fold in the BMS group. 32 up-regulated proteins and 25 down-regulated proteins were identified. (B) GO Enrichment analysis of the differentially expressed proteins (DEPs) in the subchondral bone on the ontology of biological process. (C) GO Enrichment analysis of the DEPs in the subchondral bone on the ontology of cellular component. (D) GO Enrichment analysis of the DEPs in the subchondral bone on the ontology of molecular function. (E) KEGG Enrichment analysis of the differentially expressed proteins (DEPs) in the subchondral bone. (F) WikiPathway enrichment analysis of the differentially expressed proteins (DEPs) in the subchondral bone. (The color of the circle indicates the P value, while the circle size indicates the number of DEPs)

***FABP4 regulated the expression of osteoblastic and osteoclastic markers in EPCs.***

The label-free quantitative proteomics was used to obtain information on the protein changes in the EPCs stimulated by FABP4. A total of 5,552 proteins were identified, of which 4,556 were quantifiable. When the expression of a protein was 1.5 times higher or 2/3 lower in the FABP4 group than in the control group and the p-value was <0.05, it was considered a DEP. Ultimately, 235 upregulated proteins and 202 downregulated proteins were detected (Supplemental Figure 2A). The upregulated DEPs included osteoblastic markers (Il1rn, Col6a1, Stat1, Col6a2, Col12a1, Thy1, Adamts 1, Lpl, Cdh1l, Prkaca, Postn, Il6st, Alpl, Itch, Chkb, and Stat5b) and the downregulated DEPS included osteoclastic markers (Hdac3) (Supplemental Figure 2A). The GO enrichment analysis was performed on the 437 DEPs. The data showed that these DEPs were involved in biological processes including regulation of bone mineralization and regulation of inflammatory response; cellular components including cortical microtubule cytoskeleton, cortical microtubule, and cortical cytoskeleton; and molecular function including beta-catenin binding and calcium ion binding (Supplemental Figure 2B). The KEGG enrichment analysis showed that the DEPs were significantly enriched in the PI3K-Akt signaling pathway and the WikiPathway enrichment analysis showed that the DEPs were significantly enriched in the endochondral ossification and IL-6 signaling Pathway (Supplemental Figure 2C). The regulated proteins were further validated by PRM analysis. When the expression of a protein was 1.5 times higher or 2/3 lower in the FABP4 group than in the control group and the p-value was <0.05, it was considered a significant protein. The PRM analysis demonstrated that osteoblastic markers in EPCs (Thy1, Alpl, Lpl, Il1rn, Cdh1l, Adamts 1, Il6st, Col6a2, Col6a1, Postn, and Itch) were upregulated after the stimulation of FABP4 (Supplemental Figure 2D).


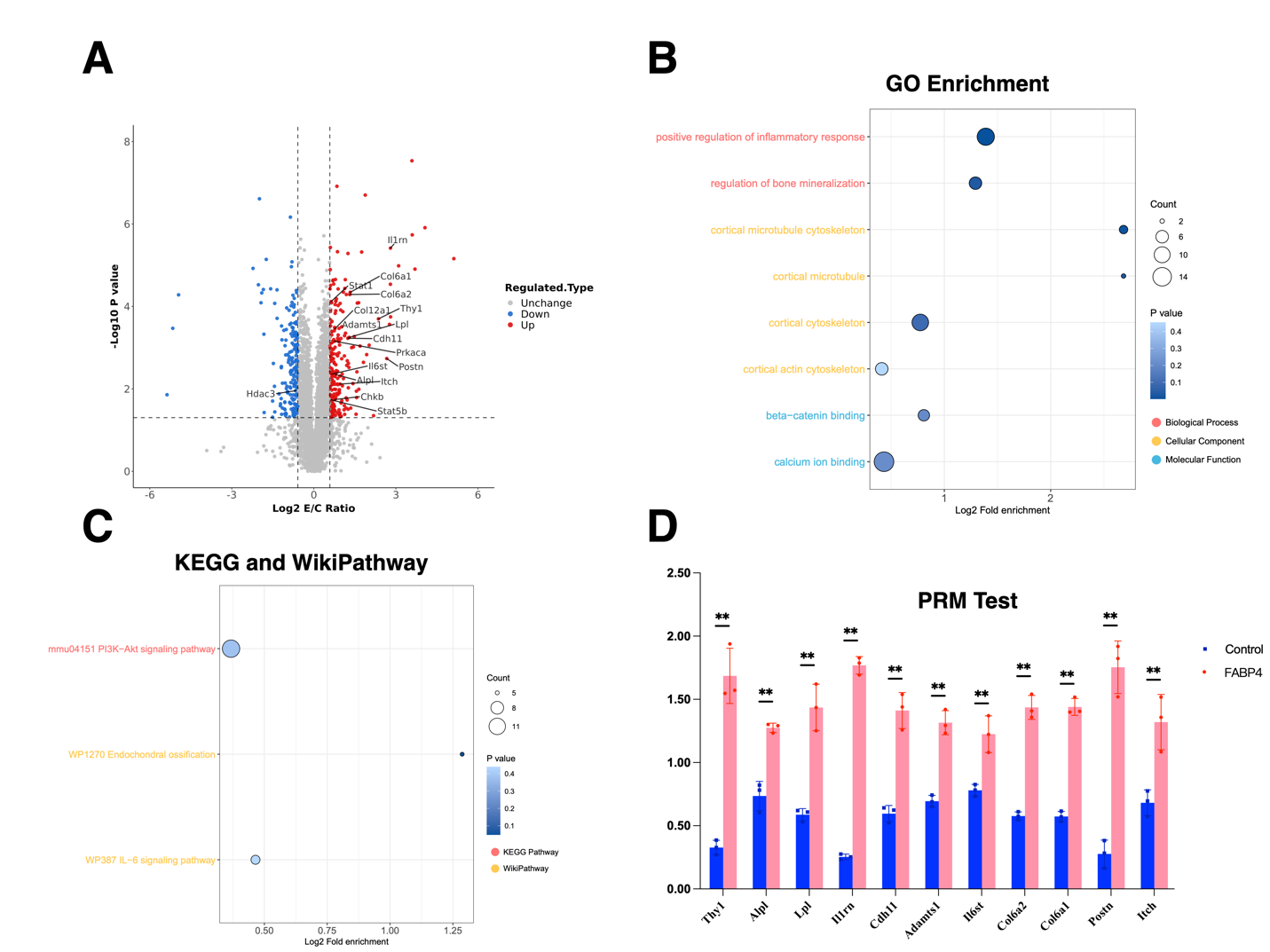


**Supplemental Figure 4. FABP4 regulated the expression of osteoblastic and osteoclastic markers in EPCs.** (A) The volcano plot illustrated the number of proteins quantified. The blue spots on the left are proteins that are down-regulated 1.5-fold in the FABP4-stimulated group and the red spots on the right are proteins that are up-regulated 1.5-fold in the FABP4-stimulated group. 235 up-regulated proteins and 202 down-regulated proteins were identified. (B) GO Enrichment analysis of the differentially expressed proteins (DEPs) of the FABP4-stimulated EPCs on the ontology of biological process, cellular component, and molecular function. (C) KEGG Enrichment analysis and WikiPathway enrichment analysis of the FABP4-stimulated EPCs. (D) The significantly expressed osteoblastic proteins between the FABP4-stimulated EPCs and unstimulated EPCs validated by the PRM analysis. (The color of the circle indicates the P value, while the circle size indicates the number of DEPs). (*n = 3, **P < 0.01)* (Student's t-test was performed for the above analysis)
